# Using Machine Learning Algorithms for Identifying Gait Parameters Suitable to Evaluate Subtle Changes in Gait in People with Multiple Sclerosis

**DOI:** 10.3390/brainsci11081049

**Published:** 2021-08-07

**Authors:** Katrin Trentzsch, Paula Schumann, Grzegorz Śliwiński, Paul Bartscht, Rocco Haase, Dirk Schriefer, Andreas Zink, Andreas Heinke, Thurid Jochim, Hagen Malberg, Tjalf Ziemssen

**Affiliations:** 1 Center of Clinical Neuroscience, Neurological Clinic, University Hospital Carl Gustav Carus, TU Dresden, Fetscherstr. 74, 01307 Dresden, Germany; katrin.trentzsch@uniklinikum-dresden.de (K.T.); paul.bartscht@uniklinikum-dresden.de (P.B.); rocco.haase@uniklinikum-dresden.de (R.H.); dirk.schriefer@uniklinikum-dresden.de (D.S.); andreas.zink@uniklinikum-dresden.de (A.Z.); 2 Institute of Biomedical Engineering, TU Dresden, Fetscherstr. 29, 01307 Dresden, Germany; paula.schumann@tu-dresden.de (P.S.); grzegorz.sliwinski@tu-dresden.de (G.Ś.); andreas.heinke@tu-dresden.de (A.H.); thurid.jochim@mailbox.tu-dresden.de (T.J.); hagen.malberg@tu-dresden.de (H.M.)

**Keywords:** multiple sclerosis, gait analysis, mobility, machine learning, feature selection

## Abstract

In multiple sclerosis (MS), gait impairment is one of the most prominent symptoms. For a sensitive assessment of pathological gait patterns, a comprehensive analysis and processing of several gait analysis systems is necessary. The objective of this work was to determine the best diagnostic gait system (DIERS pedogait, GAITRite system, and Mobility Lab) using six machine learning algorithms for the differentiation between people with multiple sclerosis (pwMS) and healthy controls, between pwMS with and without fatigue and between pwMS with mild and moderate impairment. The data of the three gait systems were assessed on 54 pwMS and 38 healthy controls. Gaussian Naive Bayes, Decision Tree, k-Nearest Neighbor, and Support Vector Machines (SVM) with linear, radial basis function (rbf) and polynomial kernel were applied for the detection of subtle walking changes. The best performance for a healthy-sick classification was achieved on the DIERS data with a SVM rbf kernel (κ = 0.49 ± 0.11). For differentiating between pwMS with mild and moderate disability, the GAITRite data with the SVM linear kernel (κ = 0.61 ± 0.06) showed the best performance. This study demonstrates that machine learning methods are suitable for identifying pathologic gait patterns in early MS.

## 1. Introduction

Multiple sclerosis (MS) is an inflammation-related chronic disease of the central nervous system that causes damage to the myelin layer of nerve fibers [1]. The manifestation of a variety of neurological symptoms may occur depending on the location of inflammatory lesions [2]. Within the classic course of the MS disease, the clinical appearance is mainly characterized by the progressive deterioration of the gait pattern [3,4]. With a prevalence of 41%, gait impairments are among the most common symptoms of the demyelinating disease [5]. Walking is a complex task involving the cooperation of several bodily functional systems, including pyramidal motor movement control and cerebellar coordination and balance [6]. Previous studies have provided evidence that gait abnormalities may well be present before they become clinically apparent [7,8,9].

Faced with the progression of MS disease and the increasing disability, the timing of treatment initiation and optimization upon treatment failure has an important impact on the course of the disease. Early initiation of therapy is essential for a more favorable disease course [10,11]. So continuous phenotyping of people with MS (pwMS) is crucial to detect early signs of progression and non-response to treatment [12,13,14]. Our concept of the implementation of an individualized, innovative management of MS is integrated in the development of digital twins [15]. Our vision is to generate and implement digital twins in the management of MS in order to improve diagnosis, treatment and management strategies as well as patient participation and compliance.

As part of such a digital twin for MS, we need to address the complexity of gait changes in pwMS and consider new methodological approaches in addition to previously used measurement tools to select the most important gait outcome parameters in the early stages of the disease for better disease monitoring. The early detection of gait changes in mild or moderate MS is helpful for the clinician to choose the best possible therapy and further therapeutic measures.

The challenge is to detect these deviations in individual cases and to differentiate them from normal variability in the general population. In practice, experts encounter several difficulties with standard clinical tests such as the 25-foot walk (T25FW) and the Expanded Disability Status Scale (EDSS) [7,16,17]. For one, there is no clear cut-off value to classify pathological gait since all measured parameters vary for each individual. For example, they strongly depend on the patient’s height, age, gender, and fitness level [18,19,20]. In addition, many of the measures are insensitive to subtle changes in gait pattern [21]. Consequently, early gait deterioration cannot be quantified and remains unnoticed until visible impairments occur. For continuous monitoring of the gait function and identification of early gait abnormalities, a variety of diagnostic tests and specific digital systems for gait analysis are necessary [22]. Digital deep phenotyping of gait can be achieved by motion capture systems based on optoelectronic stereophotogrammetry, accelerometers or force sensors which were found to be more sensitive than the T25FW for differentiating between patients and healthy controls [23,24,25,26].

Gait systems in the diagnostic process measure a wide range of outcome parameters. The identification of relevant parameters remains a challenge. In practice, only a limited selection of parameters and tests can be integrated into daily clinical routine [27]. Complementary diagnostic information may arise from wider patterns in the discarded data. Machine learning methods are suitable to detect patterns in a large amount of data. In recent years, a number of studies successfully advanced the diagnostic capability in a wide range of medical disciplines [24,28,29,30,31,32]. Jiang et al. reported a variety of medical applications where diagnostics are improved by Support Vector Machines (SVM). Such comparatively simple representatives of machine learning algorithms are performing quite well when extracting the most relevant information from complex high dimensional data [29]. Deep Learning algorithms provide a more sophisticated approach to classification problems by mimicking neuronal networks [29,31]. They perform best when trained on big data collection with thousands of samples [31,32]. Related to detailed gait analysis, Saxe et al. reviewed the current literature with appropriate metrics, devices, and algorithms [24]. Machine learning methods, namely k-Nearest Neighbors, SVM, and Neural Networks, were suitable for identifying, collecting, and rating pathological gait patterns. The evaluation of a pathological gait requires not only precise data acquisition, but also precise signal processing and feature selection [24]. Piryonesi et al. demonstrated that machine processing can predict falls and injuries in pwMS by utilizing Decision Trees and Gradient Boosted Trees [30].

This study was designed to advance machine learning in MS diagnosis and treatment through utilizing recent developments in data acquisition and data processing. Three diagnostic gait systems were compared in their capability to provide sufficient gait parameters to implement the most promising machine learning algorithms for the classification of MS in its early stages. Thereby, the machine learning approach was confronted with three tasks: first, the detection of MS, second, the detection of fatigue in pwMS and third, the differentiation of MS disability levels as measured with the EDSS. Suitable gait parameters were explored and compared to related studies for the differentiation between pwMS and healthy controls.

## 2. Materials and Methods

### 2.1. Study Design

Gait analyses were performed in a non-interventional monocentric cohort study using three different sensor-based gait systems. Each subject performed all measurements on the same day. Overall, 92 Subjects (54 pwMS and 38 healthy controls) were recruited by the MS Center Dresden (MSC) between October 2019 and February 2020. In this study, we only included patients with clinically diagnosed MS and healthy control subjects who did not require a walking aid. PwMS with an EDSS score of 1 to 4 were recruited by a physician’s neurological status assessment at the MSC of the University Hospital Dresden, Germany. Therefore, the patients as well as subjects should not be older than 65 years and they had to provide written informed consent for the study. Only pwMS who had no relapse symptoms in the course of the disease within the last four weeks prior to the assessment were included. Patients were excluded from the study if they were taking medications that influenced walking ability. These included agents with fampridine, cannabinoids, and baclofen. Furthermore, patients with an additional significant neurological or neurodegenerative disease and pwMS with limiting orthopedic impairments were not included. For the second analysis, the MS cohort was divided into two subgroups based on the presence of fatigue. The EDSS served as the criterion for this subdivision of the 54 pwMS of overall physical and cognitive fatigue. This subdivision was made to determine the feasibility of using machine learning algorithms to differentiate between pwMS with and without fatigue. The fatigue cohort consisted of 27 patients (50%), while the others showed no signs of fatigue. For the third analysis, all pwMS were divided into two groups according to their EDSS score. The definition of different disability levels were based on EDSS with mild (EDSS ≤ 2.5) and moderate disability (3.0 ≤ EDSS ≤ 4.0). The mild EDSS cohort consisted of 35 patients (65%) and moderate EDSS cohort consisted of 19 patients (35%).

Subjects were tested with GAITRite (CIR-Systems Inc., Franklin, NJ, USA) according to the Dresden Protocol for Multidimensional Gait Assessment (DMWA) [33]. The GAITRite was investigated in numerous studies and has demonstrated high reliability and validity [34,35,36]. The MSC’s walkway has a resolution of 0.6 sensors/cm^2^ and a sampling rate of 120 Hz [37]. According to the DMWA protocol, subjects had to walk twice over the 8-metre walkway at their own chosen walking speed. Next, a 2-min walk test (2MWT) was performed using the validated Mobility Lab System (APDM Inc., Portland, OR, USA) of balance and spatiotemporal gait parameters [38]. Six body worn OPAL sensors characterize the system. By processing data from the integrated accelerometers, gyroscopes, and magnetometers, the Mobility Lab provides reliable and valid gait parameters [39,40]. The sampling rate is 128 Hz [41]. For valid gait and balance parameters, the motion sensors were attached to specific parts of the body. As with other motion worn sensors, the sensor for measuring the upper sway was placed in front of the sternum 2 cm below the fossa jugularis [42]. To measure the balance of the lower torso, another sensor was placed on the lumbar spine at L5 [42,43,44]. Two further sensors were attached to the left and right wrist, 4 cm from the back of the hand [45]. The last two sensors for spatiotemporal gait parameters were placed on the forefoot [46,47]. During the 2MWT, subjects walked back and forth along a 35-m straight corridor in the MSC at a self-selected velocity. Gait endurance testing is used as an important marker in various medical fields. Originally, the Cooper 12-min walk test was developed for physical fitness and over time, shorter versions of this endurance walk test, such as the 6- and 2-min walking test have been developed [48,49]. In medicine, the 6-MWT is considered the gold standard for endurance testing [50]. However, some patients are unable to walk for more than two minutes. Therefore, the 6MWT is often too strenuous and time-consuming for cardiac patients and also for pwMS, so the 2MWT is a practical alternative in this case [49,51,52]. This is a popular and well-established walking test to obtain a detailed impression of walking ability, and there are several papers demonstrating good comparability of these two endurance walking tests [52,53,54]. Due to the high effort of 6MWT for pwMS and also limited time, space and staff resources in clinical practice, the 2MWT was favored for gait endurance testing as part of the DMWA protocol. Finally, the subjects were measured at the Institute of Biomedical Engineering at the TU Dresden using the DIERS pedogait (DIERS International GmbH, Schlangenbad, Germany). The measurement systems and methods were validated in a number of papers [55,56,57]. The pedogait system provides a functional representation of plantar pressure distribution through capacitive pressure measurement [55]. The sensor plate is integrated in the treadmill. The plate has a resolution of 1.4 sensors/cm^2^ and a sampling rate of 120 Hz [58]. Subjects were instructed to walk loosely on the treadmill facing forward. After a two-minute run-in, the measurement was performed. The measurement time lasted 6 s. Table 1 shows the gait parameters recorded by all three gait systems.

In addition to a comprehensive gait analysis, a standardized outpatient clinical neurological examination was performed as baseline. Patient-reported outcomes (PROs) were collected from study participants with MS at the end of the study. These included a self-reported measure of the impact on walking ability, using the Multiple Sclerosis Walking Scale (MSWS-12) and the Early Mobility Impairment Questionnaire (EMIQ) [59,60]. PROs are valued in the diagnosis and treatment of MS [61,62]. They are reliable and valid for the assessment of MS-related symptoms [61].

### 2.2. Basic Statistics

Quantitative population characteristics were presented as measures of central tendency (mean/median), followed by dispersion measures. Categorical characteristics were expressed as relative frequencies. Student’s *t*-test, Mann Whitney U test or chi-squared tests were used to quantify differences between pwMS and healthy controls on key characteristics. Because of the observational nature of our study and the lack of random assignment, propensity score matching was performed to balance sociodemographic characteristics between pwMS and healthy controls in case of statistically significant differences between the two groups. For this purpose, 1:1 matching without replacement was applied using propensity scores generated by logistic regression. The resulting matched data set was tested for balance by performing statistical tests for sociodemographic differences between pwMS and healthy controls (Appendix A Table A1). A matching procedure was only used for the first objective (54 pwMS vs. 38 healthy controls), as neither fatigue nor impairment affected healthy controls. The gait parameters described in the DMWA protocol are used in routine clinical practice as key parameters for the assessment of mobility changes. Therefore, these key parameters were analyzed in the further descriptive review.

### 2.3. Machine Learning Approaches

**Figure 1:**
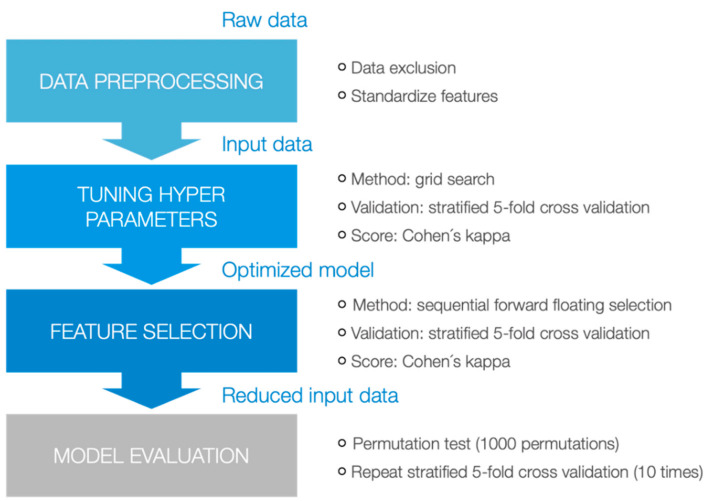
Workflow of the methodology for the application of the machine learning techniques.

To distinguish between pwMS and healthy control, six different machine learning techniques were applied: Naive Bayes, Decision Tree, k-Nearest Neighbor, and SVM with linear, radial basis function (rbf) and polynomial kernel. Ensemble learning can improve the performances of classification [63,64,65]. Therefore, a majority decision of all six models was calculated besides the evaluation of the six individual decisions. When at least three models classified a data point as pwMS, the ensemble predicted the label pwMS. The same methodology was applied to distinguish between pwMS with and without fatigue, and between mild EDSS score and moderate EDSS score. A deeper examination of the hyperparameter optimization and feature selection was undertaken by the example of the first task. The suitability of each diagnostic gait system was evaluated based on the performance of each classification task. The methodology is illustrated in Figure 1.

First, raw data were preprocessed. Gait parameters of each diagnostic gait system formed one data set (DIERS data, GAITRite data, and Mobility Lab data). These data sets were used as input features, with only metric features present in the data sets. Four participants in the Mobility Lab data set were excluded due to missing values. All features were standardized before applying the classification models.

Second, the hyperparameters were tuned. A Gaussian distribution was assumed for the Naive Bayes model. A grid search optimized the hyperparameters of the other five models. Table 2 shows the range of each hyperparameter. A stratified 5-fold cross-validation with Cohen’s kappa (κ) as evaluation score was implemented. The performance was categorized as either poor (κ < 0), slight (0 ≤ κ ≤ 0.2), fair (0.2 < κ ≤ 0.4), moderate (0.4 < κ ≤ 0.6), substantial (0.6 < κ ≤ 0.8), or almost perfect agreement (0.8 < κ < 1) [62].

Next, a sequential forward floating selection (SFFS) was applied on each classification model [66]. To find the top-n-features, the algorithm started with an empty feature space and iteratively added the feature improving Cohen’s kappa the most. After each iteration, features already contained in the subset were removed one by one until the score did not improve anymore. These steps were executed until all features were selected.

Finally, the predictions of the six classification models were evaluated. To verify how the results compare to random guessing, a permutation test was performed on each model [67]. The test assigns randomly chosen labels to data points, preserving the label distribution, and performs 1000 of these permutations to attain a *p*-value. The *p*-value of this test was calculated to estimate whether the predictions were better than random guessing. Cohen´s kappa, accuracy, sensitivity, and specificity were calculated to evaluate and compare the different classification models. The stratified 5-fold cross-validation was repeated 10 times to reduce bias when splitting the data into the folds. The machine learning analysis was performed using scikit-learn version 0.23.2 [68] and mlxtend version 0.18.0 python packages [69].

## 3. Results

### 3.1. Descriptive Analyses

For the 54 pwMS included, a median EDSS of 2 (IQR 1.5–3) was determined. 35 pwMS (65%) showed mild disability (19 pwMS moderate disability) and 27 pwMS (50%) experienced fatigue (27 pwMS without fatigue). On average, pwMS (40.3 ± 10.9) were significantly older than healthy controls (34.5 ± 13.2) (*p* = 0.002). Consequently, a 1:1 propensity score matching procedure was performed for the healthy-sick classification (first objective), with the age factor to achieve better comparability of the data. This procedure was successful, leaving no age difference between the groups (*p* = 0.96) and only dropping eight healthy controls that could not be matched. Furthermore, the matched study population (*N* = 60) demonstrated an equal sex ratio (21 females and 9 males in both the pwMS and healthy control group). A summary of the patient and disease characteristics before and after the matching procedure is provided in Table A1 in the Appendix A. A selection of key gait parameters used in clinical routine for the evaluation of mobility changes is shown in Table 3.

In the initial mean observation, the pwMS show a larger step length difference and a longer double support time compared to the healthy controls. However, checking this statement using the Mann-Whitney U-test does not result in any confirmation of a significant change in the parameters collected.

### 3.2. Machine Learning Techniques

Six classification models were used to determine the most suitable gait measurement system. Table 4 shows the optimized parameters for each classification model based on the matched collective. Different classification models were generated for each data set except the SVM with the linear kernel. The regularization C was equal (C = 0.01) to all data sets.

The classification results of each model and data set based on the matched collective are shown in Table 5. The SFFS improved the classification performance for all data sets except for Decision Tree and SVM with rbf kernel for the Mobility Lab data set. Additionally, the number of features were reduced strongly. All classification models of each data set generated a highly significant *p*-value (*p* ≤ 0.001) in the permutation test after the SFFS (apart from the Decision Tree) for all three data sets. A compilation of all top-n-relevant features for each individual data set and each classification model is presented in the Appendix A (Table A2).

The standard deviation (SD) of κ varied from 0.05 to 0.16. The largest variation of the SD (±16.6%) was observed for specificity for the GAITRite data set. The mean κ-value varied from 0.39 to 0.49 for the DIERS data set and after the SFFS. The mean κ-value varied from 0.10 to 0.28 for the GAITRite data set and after the SFFS. The mean κ-value varied from 0.02 to 0.41 for the Mobility Lab data set and after the SFFS.

The SVM with rbf kernel model (κ = 0.49 ± 0.11) was the best classification model for the DIERS data set. The k-Nearest Neighbor model (κ = 0.21 ± 0.08) was the best model for the GAITRite data set and the SVM with rbf kernel (κ = 0.41 ± 0.10) was the best model for the Mobility Lab data set. The majority decision outperformed the individual classifiers on the GAITRite data set (κ = 0.28 ± 0.09). However, it was not able to achieve better results on the other two data sets. The overall performance of the models was highest on the DIERS data set.

Moderate agreement on the DIERS data set was achieved with four models: Gaussian Naive Bayes, Decision Tree, and SVM with rbf kernel and with polynomial kernel. The Mobility Lab data set, however, only reached a single moderate agreement with SVM rbf kernel. The objective was to select the most important gait parameters that are the best to distinguish between a healthy and pathological gait pattern. For this purpose, important gait parameters were identified by determining the top-n-features by SFFS for each classifier with a score no less than moderate agreement. Gait parameters were then sorted in a frequency table, as parameters chosen by two or more classifiers were considered to be more relevant to gait classification (Table 6). Few gait parameters were used multiple times for processing. Especially the gait velocity parameter was used in three classification models of the DIERS data set and in the best possible method of the Mobility Lab data set (SVM with rbf kernel). Furthermore, step length right was used in three models of the DIERS data set along with step length left, which appeared in two of these models (Table 6).

The results of the classification of pwMS with and without fatigue are shown in Table 7. In this case, the k-Nearest Neighbor with the GAITRite data set (κ = 0.56 ± 0.05) was the best model to classify pwMS with and without fatigue. Furthermore, the resulting κ was better. The majority decision outperformed the individual classifiers on the Mobility Lab data set (κ = 0.47 ± 0.04). However, it was not able to achieve better results with the other two data sets.

The results of the classification into pwMS with mild and moderate EDSS scores are shown in Table 8. The GAITRite data set with the SVM with linear kernel (κ = 0.61 ± 0.06) generated the best performance with substantial agreement. Overall, the models had a better performance compared to the other two classification tasks. The majority decision did not achieve a better performance than the individual classification models.

## 4. Discussion

Data sets of three gait systems were compared and analyzed using machine learning methods. First, the objective was to determine which gait system provides the highest discriminatory power between pwMS and healthy controls. The DIERS system was the most successful at recognizing pwMS. The specificity was always better than the sensitivity. Consequently, the classification models are more suitable for predicting the healthy collective. Four classification models had a moderate agreement (Gaussian Naive Bayes, Decision Tree, SVM with rbf and polynomial kernel). Especially the SVM with rbf kernel performed well. The GAITRite data set was least suitable for this classification task. The best performance was a fair agreement with this data set.

Determining relevant gait parameters is important for the diagnosis of pwMS. In the clinical routine for pwMS, the interpretation of the multitude of data collected by multimodal gait analysis is often not completely possible and leads to a preselection of relevant gait parameters. The descriptive analysis of these outcome parameters shows a reduced step length difference and a longer double support time compared to other studies [9]. However, no significant changes between the healthy group and the pwMS could be found in any of the preselected outcome parameters. During clinical observation of mobility data, a certain preselection of the underlying gait parameters is done. Due to the large number of outcome parameters, it is not possible to consider them all equally in the evaluation. Machine learning methods were used to take all spatiotemporal gait parameters (as in Table 1) as a basis for the analysis without preselection.

These results are in line with previous reports investigating most relevant gait parameters to distinguish between pwMS and healthy controls. Data from a recent review by Chee et al. suggests that people with higher levels of MS-related disability have more careful and stable gait patterns compared to people with lower levels of MS-related disability [70]. The gait parameters that differentiated pwMS by their degree of disability were gait speed, step length, cadence, step time, step time variability, stance phase, and double support time. More disability was associated with shorter stride length and lower cadence [70]. In our work, especially the gait parameters of walking speed and step length were commonly selected to differentiate pwMS from healthy controls. Two models selected both sides of step length as relevant gait parameters. This suggests that both step lengths have an impact on the model, rather than leading to redundancy. Regarding pwMS, bilateral observations are an important aspect of disease monitoring since muscles degenerate at different paces [71]. However, further studies are needed validate these results. The integration of a feature selection method (SFFS) has proven to be suitable for improving the performance. However, it is important to note that the result of a SFFS is only a local optimum for a specific model. It is therefore possible that a combination of both step lengths was never inputted into the latter model.

The selection of a machine learning method depends on the data structure. This structure is often unknown and is difficult to determine due to the curse of dimensionality. Therefore, this study investigated six classification methods. These methods were used in similar classification tasks. The classification methods are simple (low train complexity) algorithms with a good interpretability. However, each method has its own advantages and disadvantages. The Naive Bayes is a simple algorithm and is suitable for small data sets, but it is often very effective in some classification tasks [72,73,74,75]. In contrary, the Naive Bayes provides a bad performance with complex data structure [73]. The present data sets consist of few data. Thus, the Naive Bayes could have provided good results here as well. The model achieved just a moderate agreement with the DIERS data set. Therefore, the present data sets could own an inherent complexity. Decision Tree is a fast adaptable classification method and is appropriate for discovering important features [76,77,78]. In the present work, the Decision Tree achieved a moderate agreement with the DIERS data set. The other two data sets only provided a slight agreement. Furthermore, the Decision Tree showed the greatest variations of the standard deviations (±16.6%) without SFFS. These classification models therefore may not be suitable for generating generally valid results. In addition, this method tends towards overfitting in contrast to other methods [76,79]. To prevent overfitting, the Decision Tree was pruned by adjusting the parameters maximum depth and minimum samples at a leaf node (hyperparameter optimization). The maximum depth was very small (Table 4) for all three data sets and the minimum samples at leaf nodes were high in the case of the DIERS data set. This indicates that the model underfit the data. In general, the trained model is too simple for the complex task. The k-Nearest Neighbor has a short training phase and is easy to use [72,80,81]. The results of the k-Nearest Neighbor models showed a fair agreement (0.21 ≤ κ ≤ 0.40) for all data sets. A disadvantage of the method is the long request time, especially when calculating distances with a high number of neighbors [72,76]. The resulting k-Nearest Neighbor models weighted features uniformly for all data sets (Table 4). Thus, important features can lose importance due to irrelevant features [72,80,81] and the method achieved just a fair agreement. The high number of Nearest Neighbors (k = 11 DIERS data set) relative to the number of samples (30 pwMS and 30 healthy control subjects) indicates that the models underfit the data. The SVM is suitable for binary classification, complex data structure and high dimensional data [76,82,83,84]. Therefore, the SVM seems suitable for the three data sets. This work investigated three kernels for the data space transformation. The SVM with a linear kernel achieved a fair agreement only with the DIERS data set. The model used a large margin for classification (small C = 0.01, Table 4). This could indicate that the model underfit the data. The SVM with a rbf kernel achieved a moderate agreement with the DIERS data set and the Mobility data set. The SVM with a polynomial kernel also achieved a moderate agreement with the DIERS data set. Nevertheless, the degree of one and the high regularization value (C = 10, Table 4) could also indicate overfitting. In summary, the Decision Tree does not seem suitable for the classification of healthy people and pwMS using gait analysis features. The SVM with rbf kernel appears more appropriate for this classification task. Overall, it is important to note that the grid search only finds a local optimum for a specific model.

Second, the detection of fatigue in pwMS was explored. The GAITRite data set achieved the best performance with κ = 0.56 and was overall the best gait system. The DIERS data set was not suitable for fatigue classification. The k-Nearest Neighbor was the best method for classifying the GAITRite and Mobility Lab data sets and achieved moderate agreement. In comparison with the healthy-sick classification, the classification models were able to achieve similar performances using gait parameters.

Third, the classification of mild and moderate EDSS score was explored in pwMS. The GAITRite data set achieved the best performance with κ = 0.61. Overall, the DIERS system was the best gait system for this task. Each model achieved a moderate agreement except for the SVM with linear kernel and the SVM with polynomial kernel. Both achieved a fair agreement. The SVM with linear kernel was the best method for classifying the GAITRite and Mobility Lab data sets and achieved substantial and moderate agreement. In comparison with the first and second classification task, the models were able to achieve better performances overall using all three gait data sets. The specificity was always better than the sensitivity. Thus, the models were able to predict mild EDSS scores especially well. In contrary to other studies, this work demonstrated that subtle gait changes could also appear for an EDSS score ≤ 4.

Previous studies reported that a majority decision can improve the performance of classification. This effect was not reflected in our data set with only the GAITRite data set in healthy-sick classification and Mobility Lab data set in fatigue classification improving on the majority decision. However, in comparison, it only achieved a fair to moderate agreement. A reason for this is the composition of the ensemble. Sagi et al. summarized two key conditions for a successful application of ensemble learning [63]. First, the methods should not be too similar in their way of decision making. Second, the quality of the performances of the individual prediction should be better than random guessing and as good as possible. This study used different methods, which are diverse in decision making. However, the range of performances of the individual models achieved slight to moderate agreement. Thus, the models could not achieve a better performance through the voting procedure.

When recognizing the limitations of this study there are reasons why the results should be generalized with caution. The small number of subjects is a disadvantage of this study. In general, more data helps to build more robust models and accurately predict the performance on new data. A total of 92 participants were included in this study. The mean age gap between pwMS und healthy control was six years. Regarding the aging process, changes in gait speed, stride length, and distance traveled occur [85,86,87]. Therefore, due to the large age difference and for better comparability of the cohorts a propensity score matching was performed. This resulted in a study cohort of 30 pwMS and 30 healthy controls. No test data set was used due the small data size. Therefore, the results could have a positive bias. Stratified 5-fold cross-validation was used for grid search, SFFS and performance evaluation in order to make the results generally valid [88]. However, a cross validation score could be obtained by chance, the split of the folds being a significant issue [89]. Thus, the cross-validation was repeated 10 times and preceded by a permutation test. These defined methods are sufficient to evaluate the results. The results showed that all classification models for each data set performed better than random guessing (*p* ≤ 0.001) after the SFFS except the Decision Tree.

Furthermore, it must be considered that each measurement system is based on different physical measurement principles. In this work, gait parameters were obtained from the processing of resistive pressure sensors [35], accelerometer, gyroscope and magnetometer sensors [38] and capacitive pressure sensors [56]. Gait changes in pwMS affect not only spatiotemporal parameters, but also kinematics and kinetics. Indeed, spatiotemporal parameters, especially in pwMS with mild disabilities are often similar to those in healthy individuals, and the differences only become visible with special processing techniques. An evaluation of video-based data was not possible for precise classification of gait patterns, even though it is continuously developed and proved to be a very reliable tool for gait analysis [90].

Due to the varying degrees of gait abnormalities in pwMS, it seems useful to confidently classify the types of mobility impairments and evaluate the applicability of machine learning methods to support the phenotyping of pwMS. The accurate classification of the different walking impairments could then be used to characterize the MS phenotype. Continuous characterization of the MS phenotype will allow more specific treatment decisions to be made by the clinicians providing treatment and an early counteracting of disability progression.

## 5. Conclusions

This work demonstrated that the DIERS system was the most appropriate gait system for healthy-sick classification among the examined devices. Velocity and step length were especially relevant for this classification task. The GAITRite system was suitable for disease monitoring though the detection of fatigue and the differentiation of mild and moderate EDSS score. In addition, the differentiation between mild and moderate EDSS score achieved the highest performance in this study with a κ = 0.6. The k-Nearest Neighbor and the SVM were suitable to discriminate subtle gait changes. Further investigation of other analyzing methods in the field of hyperparameter optimization and feature selection could improve the performances and generalize the models. For future work, it is relevant to analyze a larger pwMS cohort with different MS courses using the algorithms presented here.

Machine learning strategies enable the integration and visualization of gait parameters collected in routine clinical practice. Based on this data, model calculations can be used to quantify certain phenotypes and generate algorithms from which more specific and also more individualized treatment guidelines could be derived. Regarding the increasing amount of data, it is important to increasingly include machine learning strategies into the phenotyping of MS to provide an individualized comprehensive view of gait changes as part of the development of innovative disease management concepts such as digital twins for MS.

## Figures and Tables

**Table 1:** Recorded gait parameters of three diagnostic gait analysis systems; the unit of each gait parameters is shown in parentheses. The parameters have been recorded and merged for the left and right sides (L/R); The parameters are presented as mean [mean] or standard deviation [SD]; ( )—dimensionless values; COP = center of pressure; GCT = gait cycle time; HH = heel to heel.

DIERS	GAITRite	Mobility Lab
Bipedale Phase (%GCT) [mean]	Ambulation Time (s) [mean]	Duration (s)
Cadence (steps/min) [mean]	Cadence (steps/min) [mean]	Lower Limb—Cadence L/R (steps/min) [mean]/[SD]
COP-Deflection lateral L/R (cm) [mean]	Cycle Time Differential (s)	Lower Limb—Circumduction L/R (cm) [mean]/[SD]
Distance (cm) [mean]	Cycle Time L/R (s) [mean]	Lower Limb—Double Support L/R (%GCT) [mean]/[SD]
Foot Rotation L/R (degrees) [mean]	Distance (cm) [mean]	Lower Limb—Elevation at Midswing L/R (cm) [mean]/[SD]
Forefoot L/R (% Stance Phase) [mean]	Double Supp. Time L/R (s)/(%GCT) [mean]/[SD]	Lower Limb—Foot Strike Angle L/R (degrees) [mean]/[SD]
Loading Response L/R (%GCT) [mean]	Double Support Load Time L/R (s)/ (%GCT) [mean]	Lower Limb—Gait Cycle Duration L/R (s) [mean]/[SD]
Midfoot L/R (% Stance Phase) [mean]	Double Support Unload Time L/R (s)/(%GCT) [mean]	Lower Limb—Gait Speed L/R (m/s) [mean]/[SD]
Pre-Swing Phase L/R (%GCT) [mean]	Functional Amb. Profile ( )	Lower Limb—Lateral Step Variability L/R (cm)
Rearfoot L/R (% Stance Phase) [mean]	Heel Off On Perc L/R (s) [mean]	Lower Limb—N (#)
Single Support L/R (%GCT) [mean]	Heel Off On Time L/R (s) [mean]/[SD]	Lower Limb—Single Limb Support L/R (%GCT) [mean]/[SD]
Stance Phase L/R (%GCT) [mean]	HH-Base Support L/R (cm) [mean]/[SD]	Lower Limb—Stance L/R (%GCT) [mean]/[SD]
Step Length L/R (cm) [mean]	Normalized Velocity (cm/s) [mean]	Lower Limb—Step Duration L/R (s) [mean]/[SD]
Step Time L/R (ms) [mean]	Single Supp. Time L/R (s)/(%GCT) [mean]/[SD]	Lower Limb—Stride Length L/R (m) [mean]/[SD]
Step Width (cm) [mean]	Stance Time L/R (s)/(%GCT) [mean]/[SD]	Lower Limb—Swing L/R (%GCT) [mean]/[SD]
Stride Length (cm) [mean]	Step Count ( )	Lower Limb—Terminal Double Support L/R (%GCT) [mean]/[SD]
Stride Time (ms) [mean]	Step Extremity L/R (ratio)	Lower Limb—Toe Off Angle L/R (degrees) [mean]/[SD]
Swing Phase L/R (%GCT) [mean]	Step Length Differential (cm)	Lower Limb—Toe Out Angle L/R (degrees) [mean]/[SD]
Velocity (km/h) [mean]	Step Length L/R (cm) [mean]/[SD]	Lumbar/Trunk—Coronal Range of Motion (degrees) [mean]/[SD]
Walk Track anterior/posterior Position (mm) [SD]	Step Time Differential (s)	Lumbar/Trunk—Sagittal Range of Motion (degrees) [mean]/[SD]
Walk Track lateral Position (mm) [SD]	Step Time L/R (s) [mean]/[SD]	Lumbar/Trunk—Transverse Range of Motion (degrees) [mean]/[SD]
	Stride Length L/R (cm) [mean]/[SD]	Turns—Angle (degrees) [mean]/[SD]
	Stride Time L/R (s) [SD]	Turns—Duration (s) [mean]/[SD]
	Stride Velocity L/R (cm/s) [mean]/[SD]	Turns—N ( )
	Swing Time L/R (s)/(%GCT) [mean]/[SD]	Turns—Steps in Turn ( ) [mean]/[SD]
	Toe In/Out L/R (degrees) [mean]	Turns—Turn Velocity (degrees/s) [mean]/[SD]
	Velocity (cm/s) [mean]	Upper Limb—Arm Range of Motion L/R (degrees) [mean]/[SD]
		Upper Limb—Arm Swing Velocity L/R (degrees/s) [mean]/[SD]

**Table 2:** Range of hyperparameters optimized with grid search; SVM = Support Vector Machine; rbf = radial basis function.

Method	Hyperparameter	Min	Max	Step Size	Scale
Decision Tree	Criterion: ‘gini’ or ‘entropy’	-	-	-	-
	Maximum depth	2	7	1	linear
	Minimum samples at a leaf node	5	20	1	linear
k-Nearest Neighbor	Weights: ‘uniform’ or ‘distance’	-	-	-	-
	Distance metric: ‘euclidean’ or ‘manhattan’	-	-	-	-
	Numbers of neighbors k	2	22	1	linear
SVM (linear kernel)	Regularization C	0.01	10	10	logarithmic
SVM (rbf kernel)	Regularization C	1	10	1	linear
	Kernel coefficient gamma	0.01	0.1	0.01	linear
SVM (polynomial kernel)	Regularization C	0.1	10	10	logarithmic
	Kernel coefficient gamma	0.01	0.1	0.01	linear
	Degree	1	10	1	linear

**Table 3:** Selected gait parameters for people with MS and healthy controls (*N* = 60). Key parameters used in clinical routine according our DMWA protocol [33]; MS = multiple sclerosis; HC = healthy controls; EMIQ = Early Mobility Impairment Questionnaire; MSWS = Multiple Sclerosis Walking Scale; GCT = Gait Cycle Time; L = left; R = right; standard deviation = SD; data in mean ± SD; ( )—dimensionless values; *p*-value via Mann-Whitney U-Test for differences between groups.

Outcome Variable	MS (*N* = 30)	HC (*N* = 30)	*p*
	**GAITRite**		
Velocity (m/s)	1.3 ± 0.1	1.3 ± 0.2	0.652
Step length difference (cm)	2.0 ± 1.7	1.4 ± 1.2	0.107
Step time difference (ms)	11.9 ± 8.9	8.0 ± 7.0	0.079
Base of support (cm) L	9.2 ± 2.7	9.4 ± 2.1	0.756
Base of support (cm) R	9.2 ± 2.6	9.4 ± 2.2	0.393
Functional ambulation profile ( )	97.5 ± 3.1	96.8 ± 3.9	
	**Mobility Lab**		
Gait speed (m/s) L	1.4 ± 0.1	1.4 ± 0.2	0.177
Gait speed (m/s) R	1.4 ± 0.1	1.4 ± 0.1	0.093
Double support (%GCT) L	18.7 ± 2.9	17.6 ± 2.2	0.170
Double support (% GCT) R	18.7 ± 2.9	17.7 ± 2.2	0.167
Stance (%GCT) L	59.5 ± 1.7	58.9 ± 1.0	0.131
Stance (% GCT) R	59.2 ± 1.5	58.7 ± 1.4	0.225
	**Patient reported outcomes**		
EMIQ	11.0 ± 13.1		
MSWS-12	11.0 ± 17.4		

**Table 4:** Results of the hyperparameter optimization for each data set (matched collective); SVM = Support Vector Machine; rbf = radial basis function.

	Parameter	DIERS Data Set	GAITRite Data Set	Mobility Lab Data Set
Decision Tree	Criterion	gini	entropy	entropy
	Maximum depth	2	2	3
	Minimum samples at a leaf node	18	5	9
k-Nearest Neighbor	Weights	uniform	uniform	uniform
	Distance metric	euclidean	manhattan	euclidean
	Numbers of neighbors k	11	2	9
SVM (linear kernel)	Regularization C	0.01	0.01	0.01
SVM (rbf kernel)	Regularization C	3	3	1
	Kernel coefficient gamma	0.04	0.01	0.06
SVM (polynomial kernel)	Regularization C	1	0.1	0.1
	Kernel coefficient gamma	0.08	0.03	0.01
	Degree	1	3	1

**Table 5:** Performance of the six classification models on the detection of MS in 60 subjects (matched collective). The values are presented as mean ± standard deviation across 5-fold cross-validation repetition. In addition, the majority decision of all six models is shown. A majority decision for a positive label occurs when at least three models (≥3) predicted the positive class. ^a^ Best overall performance per category for each data set; SVM = Support Vector Machine; rbf = radial basis function; SFFS = sequential forward floating selection; *p*-value via permutation test.

		No. Features	Cohen’S Kappa	Accuracy (%)	Sensitivity (%)	Specificity (%)	*p*
**DIERS data set**							
Gaussian Naive Bayes	Without SFFS	33	0.26 ± 0.05	63.2 ± 2.5	51.0 ± 2.7	75.3 ± 3.2	0.025
	With SFFS	11	0.46 ± 0.06	73.2 ± 2.8	64.3 ± 3.2	82.0 ± 3.6	0.001
Decision Tree	Without SFFS	33	0.24 ± 0.06	62.0 ± 2.9	62.0 ± 3.9	62.0 ± 5.7	0.085
	With SFFS	1	0.43 ± 0.05	71.3 ± 2.5	66.0 ± 2.1	76.7 ± 3.8	0.002
k-Nearest Neighbor	Without SFFS	33	0.23 ± 0.06	61.3 ± 3.0	39.0 ± 4.5	83.7 ± 4.0	0.020
	With SFFS	5	0.40 ± 0.10	69.8 ± 4.8	62.7 ± 6.2	77.0 ± 5.1	0.001
SVM (linear kernel)	Without SFFS	33	0.26 ± 0.07	63.2 ± 3.6	56.0 ± 5.2	70.3 ± 5.1	0.002
	With SFFS	28	0.39 ± 0.07	69.7 ± 3.6	63.0 ± 4.8	76.3 ± 6.4	0.001
SVM (rbf kernel)	Without SFFS	33	0.20 ± 0.10	60.0 ± 4.8	59.0 ± 5.2	61.0 ± 6.7	0.008
	With SFFS	8	0.49 ± 0.11 ^a^	74.5 ± 5.5 ^a^	67.0 ± 6.2 ^a^	82.0 ± 6.1 ^a^	0.001 ^a^
SVM (polynomial kernel)	Without SFFS	33	0.24 ± 0.09	61.8 ± 4.5	55.7 ± 6.9	68.0 ± 7.1	0.001
	With SFFS	13	0.41 ± 0.06	70.3 ± 3.2	63.0 ± 4.0	77.7 ± 5.5	0.001
Majority decision (≥3)	With SFFS	-	0.49 ± 0.08	74.5 ± 3.9	69.7 ± 3.7	79.3 ± 5.8	-
**GAITRite data set**							
Gaussian Naive Bayes	Without SFFS	76	0.01 ± 0.09	50.3 ± 4.7	70.3 ± 5.1	30.3 ± 5.3	0.141
	With SFFS	8	0.19 ± 0.10	59.7 ± 5.2	63.3 ± 6.1	56.0 ± 6.6	0.001
Decision Tree	Without SFFS	76	−0.02 ± 0.12	49.0 ± 5.9	35.7 ± 11.8	62.3 ± 16.6	0.170
	With SFFS	3	0.10 ± 0.16	55.2 ± 4.8	61.1 ± 8.6	46.8 ± 7.2	0.008
k-Nearest Neighbor	Without SFFS	76	0.11 ± 0.07	55.5 ± 3.7	26.3 ± 4.3	84.7 ± 5.9	0.116
	With SFFS	31	0.21 ± 0.08	60.7 ± 4,0	38.0 ± 4.5	83.3 ± 5.4	0.001
SVM (linear kernel)	Without SFFS	76	0.14 ± 0.12	57.2 ± 5.8	60.7 ± 8.6	53.7 ± 6.2	0.120
	With SFFS	18	0.16 ± 0.13	58.0 ± 6.7	61.3 ± 5.0	54.7 ± 10.6	0.001
SVM (rbf kernel)	Without SFFS	76	0.08 ± 0.07	54.2 ± 3.7	52.0 ± 4.5	56.3 ± 6.9	0.216
	With SFFS	34	0.20 ± 0.09	59.8 ± 4.5	50.7 ± 5.2	69.0 ± 6.7	0.001
SVM (polynomial kernel)	Without SFFS	76	0.16 ± 0.09	58.2 ± 4.6	93.0 ± 4.6	23.3 ± 7.5	0.005
	With SFFS	10	0.17 ± 0.11	58.7 ± 5.4	70.7 ± 8.1	46.7 ± 13.1	0.001
Majority decision (≥3)	With SFFS	-	0.28 ± 0.09 ^a^	63.8 ± 4.4 ^a^	67.3 ± 7.0 ^a^	60.3 ± 4.6 ^a^	-
**Mobility Lab data set**							
Gaussian Naive Bayes	Without SFFS	93	0.10 ± 0.06	55.1 ± 3.0	53.8 ± 6.1	56.4 ± 2.8	0.492
	With SFFS	15	0.36 ± 0.08	67.9 ± 3.9	71.7 ± 5.6	63.9 ± 4.9	0.001
Decision Tree	Without SFFS	93	0.08 ± 0.09	54.0 ± 4.4	53.1 ± 10.0	55.0 ± 6.6	0.198
	With SFFS	41	0.08 ± 0.08	54.2 ± 4.0	52.8 ± 11.7	55.7 ± 7.6	0.007
k-Nearest Neighbor	Without SFFS	93	0.08 ± 0.07	53.3 ± 3.5	17.2 ± 6.9	90.7 ± 3.5	0.100
	With SFFS	9	0.33 ± 0.06	66.1 ± 3.2	55.9 ± 4.5	76.8 ± 5.1	0.001
SVM (linear kernel)	Without SFFS	93	0.01 ± 0.07	50.5 ± 3.7	48.6 ± 7.4	52.5 ± 6.1	0.495
	With SFFS	5	0.20 ± 0.07	60.0 ± 3.4	59.7 ± 5.2	60.4 ± 5.4	0.001
SVM (rbf kernel)	Without SFFS	93	0.20 ± 0.06	60.5 ± 3.0	90.3 ± 3.6	29.6 ± 5.1	0.004
	With SFFS	24	0.41 ± 0.10 ^a^	70.4 ± 5.0 ^a^	77.9 ± 6.5 ^a^	62.5 ± 7.2 ^a^	0.001 ^a^
SVM (polynomial kernel)	Without SFFS	93	0.00 ± 0.07	50.4 ± 3.3	80.3 ± 5.2	19.3 ± 3.8	0.035
	With SFFS	12	0.02 ± 0.07	51.6 ± 3.3	80.0 ± 7.8	22.1 ± 3.7	0.001
Majority decision (≥3)	With SFFS	-	0.34 ± 0.08	67.2 ± 3.8	83.8 ± 6.5	50.0 ± 7.1	-

**Table 6:** List of features for classification models with moderate agreement for each data set (matched collective). The gait parameters are given in their mean value. If the standard deviation of the gait parameter is meant, it is explicitly followed by [SD].

Features	No. of Uses
**DIERS data set (Gaussian Naive Bayes, Decision Tree, SVM with rbf and polynomial kernel)**	
Single Support L, Step Length R, Velocity	3
Foot Rotation L, Loading Response L, Pre-Swing Phase L, Step Length L, Stride Length, Stride Time, Walk Track anterior/posterior Position [SD]	2
Cadence, COP-Deflection lateral L, COP-Deflection lateral R, Foot Rotation R, Midfoot L, Midfoot R, Pre-Swing Phase R, Rearfoot L, Stance Phase R, Swing Phase R	1
**Mobility Lab data set (SVM with rbf kernel)**	
Lower Limb—Double Support R, Lower Limb—Foot Strike Angle R [SD], Lower Limb—Gait Speed L, Lower Limb—Gait Speed R, Lower Limb—Lateral Step Variability L, Lower Limb—Single Limb Support R, Lower Limb—Stance L, Lower Limb—Stride Length L, Lower Limb—Terminal Double Support R, Lower Limb—Toe Off Angle L, Lower Limb—Toe Off Angle L [SD], Lower Limb—Toe Off Angle R, Lower Limb—Toe Off Angle R [SD], Lower Limb—Toe Out Angle R [SD], Lumbar—Coronal Range of Motion [SD], Lumbar—Sagittal Range of Motion, Lumbar—Sagittal Range of Motion [SD], Trunk—Coronal Range of Motion [SD], Trunk—Transverse Range of Motion [SD], Turns—Turn Velocity [SD], Upper Limb—Arm Range of Motion L, Upper Limb—Arm Range of Motion L [SD], Upper Limb—Arm Swing Velocity L [SD], Upper Limb—Arm Swing Velocity R [SD]	1

**Table 7:** Performance of the six classification models on the detection of fatigue in people with multiple sclerosis (*n* = 54) after hyperparameter optimization and feature selection. In addition, the majority decision of all six models is shown. A majority decision for a positive label occurs when at least three models (≥3) predicted the positive class. The values are presented as mean ± standard deviation across 10 times 5-fold cross-validation repetition. ^a^ Best overall performance per category for each data set; SVM = Support Vector Machine; rbf = radial basis function; *p*-value via permutation test.

	Performance	DIERS Data Set	GAITRite Data Set	Mobility Lab Data Set
Gaussian Naive Bayes	Cohen’s kappa:	0.06 ± 0.12	0.35 ± 0.08	0.11 ± 0.12
	Accuracy (%):	53.1 ± 5.9	67.6 ± 4.0	55.7 ± 6.2
	*p*:	0.009	0.001	0.001
Decision Tree	Cohen’s kappa:	0.24 ± 0.04 ^a^	0.29 ± 0.13	0.36 ± 0.03
	Accuracy (%):	62.2 ± 1.8 ^a^	64.6 ± 6.5	67.9 ± 1.5
	*p*:	0.050	0.001	0.031
k-Nearest Neighbor	Cohen’s kappa:	0.18 ± 0.05	0.56 ± 0.05 ^a^	0.42 ± 0.10
	Accuracy (%):	58.9 ± 2.4	78.1 ± 2.7 ^a^	71.1 ± 4.9
	*p*:	0.002	0.001 ^a^	0.001
SVM (linear kernel)	Cohen’s kappa:	−0.01 ± 0.10	0.34 ± 0.06	0.32 ± 0.07
	Accuracy (%):	49.4 ± 4.9	67.0 ± 3.2	65.8 ± 3.4
	*p*:	0.004	0.001	0.001
SVM (rbf kernel)	Cohen’s kappa:	0.10 ± 0.11	0.36 ± 0.12	0.41 ± 0.06
	Accuracy (%):	55.2 ± 5.6	67.8 ± 5.8	70.4 ± 3.1
	*p*:	0.013	0.001	0.001
SVM (polynomial kernel)	Cohen’s kappa:	0.09 ± 0.11	0.35 ± 0.05	−0.02 ± 0.06
	Accuracy (%):	54.6 ± 5.4	67.6 ± 2.5	48.7 ± 3.1
	*p*:	0.001	0.001	0.001
Majority decision (≥3)	Cohen’s kappa:	0.13 ± 0.11	0.50 ± 0.04	0.47 ± 0.04 ^a^
	Accuracy (%):	56.5 ± 5.3	74.8 ± 1.8	73.2 ± 2.1 ^a^

**Table 8:** Performance of the six classification models on the detection of mild EDSS or moderate EDSS in people with multiple sclerosis (*n* = 54) after hyperparameter optimization and feature selection. In addition, the majority decision of all six models is shown. A majority decision for a positive label occurs when at least three models (≥3) predicted the positive class. The values are presented as mean ± standard deviation across 10 times 5-fold cross-validation repetition. Sensitivity and specificity were used instead of the accuracy due to imbalanced data (65% mild, 35% moderate). ^a^ Best overall performance per category for each data set; SVM = Support Vector Machine; rbf = radial basis function; *p*-value via permutation test.

	Performance	DIERS Data Set	GAITRite Data Set	Mobility Lab Data Set
Gaussian Naive Bayes	Cohen’s kappa:	0.57 ± 0.03 ^a^	0.35 ± 0.12	0.31 ± 0.08
	Sensitivity (%):	60.5 ± 2.8 ^a^	56.8 ± 6.5	57.4 ± 5.8
	Specificity (%):	93.1 ± 1.5 ^a^	77.7 ± 6.7	74.1 ± 5.0
	*p*:	0.001 ^a^	0.001	0.001
Decision Tree	Cohen’s kappa:	0.53 ± 0.11	0.43 ± 0.05	0.47 ± 0.03
	Sensitivity (%):	63.7 ± 9.4	66.3 ± 6.7	60.5 ± 2.8
	Specificity (%):	88.0 ± 5.5	77.1 ± 3.0	85.3 ± 0.0
	*p*:	0.001	0.002	0.001
k-Nearest Neighbor	Cohen’s kappa:	0.52 ± 0.06	0.40 ± 0.08	0.39 ± 0.11
	Sensitivity (%):	51.6 ± 4.8	48.4 ± 6.5	47.4 ± 7.8
	Specificity (%):	95.7 ± 2.4	88.6 ± 4.7	89.1 ± 5.6
	*p*:	0.001	0.001	0.001
SVM (linear kernel)	Cohen’s kappa:	0.37 ± 0.09	0.61 ± 0.06 ^a^	0.48 ± 0.09 ^a^
	Sensitivity (%):	51.6 ± 7.4	70.0 ± 5.0 ^a^	67.9 ± 7.2 ^a^
	Specificity (%):	83.7 ± 3.8	89.7 ± 2.8 ^a^	80.3 ± 4.6 ^a^
	*p*:	0.001	0.001 ^a^	0.001 ^a^
SVM (rbf kernel)	Cohen’s kappa:	0.44 ± 0.02	0.18 ± 0.09	0.20 ± 0.07
	Sensitivity (%):	41.1 ± 2.2	17.4 ± 5.6	23.7 ± 6.2
	Specificity (%):	97.1 ± 1.3	97.7 ± 2.3	93.5 ± 2.3
	*p*:	0.001	0.001	0.001
SVM (polynomial kernel)	Cohen’s kappa:	0.40 ± 0.03	0.45 ± 0.11	0.15 ± 0.03
	Sensitivity (%):	36.8 ± 4.3	61.6 ± 7.5	13.2 ± 2.8
	Specificity (%):	97.7 ± 1.8	82.3 ± 5.8	98.8 ± 1.5
	*p*:	0.001	0.001	0.018
Majority decision (≥3)	Cohen’s kappa:	0.55 ± 0.06	0.60 ± 0.05	0.47 ± 0.09
	Sensitivity (%):	54.2 ± 5.0	69.5 ± 3.3	54.2 ± 5.0
	Specificity (%):	96.3 ± 2.4	88.9 ± 4.1	89.7 ± 5.4

## Data Availability

The data presented in this study are available on request from the corresponding author. The data are not publicly available due to patient confidentiality.

## Appendix A

**Table A1:** Characterization of people with multiple sclerosis (MS) and healthy controls (HC), respectively, before and after propensity score matching (*N* = 92); SD = Standard Deviation; EDSS = Expanded Disability Status Scale; RRMS = relapsing–remitting MS; PPMS = primary progressive MS; ^b^ *p*-value via *t*-test for differences between groups; ^c^ *p*-value via chi-squared test for differences between groups.

			Unmatched Set (*N* = 92)			Matched Set (*N* = 60)		
			MS (*N* = 54)	HC (*N* = 38)	*p*	MS (*N* = 30)	HC (*N* = 30)	*p*
Mean age in years (mean ± SD)			40.3 ± 10.9	34.0 ± 13.3	0.002 ^b^	37.1 ± 12.5	36.9 ± 13.5	0.961 ^b^
Gender	Female N (%)		35 (64.8%)	23 (60.5%)	0.675 ^c^	21 (70.0%)	21 (70.0%)	0.999 ^c^
	Male N (%)		19 (35.2%)	15 (39.5%)		9 (30.0%)	9 (30.0%)	
Duration of disease in years (mean ± SD)			8.1 ± 6.0			7.1 ± 5.4		
EDSS (median) EDSS (Interquartile range)			2 1.5–3.0			1.5 1.5–2.6		
Disease Course N (%)		RRMS	52 (96.3%)			29 (96.7%)		
		PPMS	2 (3.7%)			1 (3.3%)		

**Table A2:** List of all top-n-relevant features for each individual data set (matched collective) and classification model. The parameters have been recorded and merged for the left and right sides (L/R). The gait parameters are given in their mean value. If the standard deviation of the gait parameter is meant, it is explicitly followed by [SD]; SVM = Support Vector Machine; rbf = radial basis function.

	No. Features	Features
**DIERS data set**		
Gaussian Naive Bayes	11	COP-Deflection lateral R, Foot Rotation L, Foot Rotation R, Pre-Swing Phase R, Single Support L, Step Length L, Step Length R, Stride Length, Stride Time, Velocity, Walk Track anterior/posterior Position [SD]
Decision Tree	1	Velocity
k-Nearest Neighbor	5	Rearfoot L, Stance Phase R, Step Time L, Stride Length, Stride Time
SVM (linear kernel)	28	Bipedale Phase, Cadence, COP-Deflection lateral L, Foot Rotation L, Forefoot R, Loading Response L, Loading Response R, Midfoot L, Midfoot R, Pre-Swing Phase L, Pre-Swing Phase R, Rearfoot L, Rearfoot R, Single Support L, Single Support R, Stance Phase L, Stance Phase R, Step Length L, Step Length R, Step Time L, Step Width, Stride Length, Stride Time, Swing Phase L, Swing Phase R, Velocity, Walk Track anterior/posterior Position [SD], Walk Track lateral Position [SD]
SVM (rbf kernel)	8	Cadence, Foot Rotation L, Loading Response L, Pre-Swing Phase L, Single Support L, Step Length R, Velocity, Walk Track anterior/posterior Position [SD]
SVM (polynomial kernel)	13	COP-Deflection lateral L, Loading Response L, Midfoot L, Midfoot R, Pre-Swing Phase L, Rearfoot L, Single Support L, Stance Phase R, Step Length L, Step Length R, Stride Length, Stride Time, Swing Phase R
**GAITRite data set**		
Gaussian Naive Bayes	8	Cycle Time Differential, Double Support Load Time R (%GC), HH Base Support L, Stance Time L, Step Extremity R, Step Time Differential, Stride Velocity L [SD], Swing Time L
Decision Tree	3	Stance Time L (%GC), Step Count, Swing Time R
k-Nearest Neighbor	31	Ambulation Time, Cadence, Cycle Time L, Distance, Double Supp. Time L (%GC), Double Supp. Time R (%GC), Double Supp. Time L [SD], Double Supp Time R [SD], Double Supp. Time L, Double Supp. Time R, Double Support Load Time L (%GC), Double Support Load Time L, Double Support Unload Time L (%GC), Double Support Unload Time R (%GC), Double Support Unload Time R, HH Base Support L, HH Base Support R, Single Supp. Time L (%GC), Single Supp. Time R (%GC), Stance Time L (%GC), Stance Time R [SD], Step Count, Step Extremity L, Step Extremity R, Step Length L [SD], Step Time L [SD], Step Time R [SD], Stride Time R [SD], Stride Velocity R, Swing Time L (%GC), Swing Time R
SVM (linear kernel)	18	Double Supp. Time L (%GC), Double Supp. Time R (%GC), Double Support Load Time L (%GC), Double Support Unload Time R (%GC), Double Support Unload Time R, Single Supp. Time L (%GC), Single Supp. Time R (%GC), Stance Time R (%GC), Stance Time L, Step Extremity R, Step Length L, Step Length R, Step Time Differential, Stride Length L [SD], Stride Length L, Stride Length R, Swing Time R (%GC), Swing Time R [SD]
SVM (rbf kernel)	34	Distance, Double Supp. Time L (%GC), Double Supp. Time R (%GC), Double Supp. Time R [SD], Double Supp. Time L, Double Supp. Time R, Double Support Load Time L (%GC), Double Support Load Time L, Double Support Load Time R, Double Support Unload Time L (%GC), Double Support Unload Time R (%GC), Double Support Unload Time R, Heel Off On Perc R, HH Base Support L, HH Base Support R, Single Supp. Time L (%GC), Single Supp. Time R (%GC), Stance Time L (%GC), Stance Time R (%GC), Step Count, Step Length Differential, Step Length L, Step Length R, Step Time Differential, Stride Length L [SD], Stride Length L, Stride Length R, Stride Velocity L, HH Base Support R [SD], Swing Time L (%GC), Swing Time R (%GC), Swing Time R, Toe In / Out R, Velocity
SVM (polynomial kernel)	10	Distance, Double Supp. Time L, Double Support Unload Time L (%GC) L, Heel Off On Perc R, Heel Off On L [SD], Step Time Differential, Stride Velocity L, Stride Velocity L [SD], Swing Time L, Toe In / Out R
**Mobility Lab data set**		
Gaussian Naive Bayes	15	Lower Limb—Double Support L (%GCT), Lower Limb—Double Support R (%GCT), Lower Limb—Foot Strike Angle R, Lower Limb—Stance R (%GCT), Lower Limb—Terminal Double Support R (%GCT) [SD], Lower Limb—Toe Off Angle L, Lower Limb—Toe Off Angle R, Lumbar—Sagittal Range of Motion, Trunk—Coronal Range of Motion [SD], Trunk—Sagittal Range of Motion, Trunk—Transverse Range of Motion, Turns—N, Turns—Steps in Turn, Turns—Turn Velocity, Upper Limb—Arm Range of Motion L
Decision Tree	41	Duration, Lower Limb—Cadence L, Lower Limb—Cadence R, Lower Limb—Circumduction L, Lower Limb—Circumduction L [SD], Lower Limb—Circumduction R, Lower Limb—Elevation at Midswing L, Lower Limb—Elevation at Midswing R, Lower Limb—Elevation at Midswing R [SD], Lower Limb—Foot Strike Angle L, Lower Limb—Foot Strike Angle L [SD], Lower Limb—Foot Strike Angle R, Lower Limb—Foot Strike Angle R [SD], Lower Limb—Gait Cycle Duration L, Lower Limb—Gait Cycle Duration L [SD], Lower Limb—Gait Cycle Duration R, Lower Limb—Gait Cycle Duration R [SD], Lower Limb—Gait Speed L, Lower Limb—Gait Speed R, Lower Limb—Lateral Step Variability L, Lower Limb—Lateral Step Variability R, Lower Limb—N, Lower Limb—Single Limb Support L (%GCT) [SD], Lower Limb—Stance L (%GCT), Lower Limb—Stance L (%GCT) [SD], Lower Limb—Stance R (%GCT) [SD], Lower Limb—Step Duration L, Lower Limb—Step Duration L [SD], Lower Limb—Step Duration R, Lower Limb—Step Duration R [SD], Lower Limb—Stride Length L, Lower Limb—Stride Length L [SD], Lower Limb—Toe Off Angle L, Lower Limb—Toe Off Angle L [SD], Lower Limb—Toe Off Angle R, Lower Limb—Toe Off Angle R [SD], Lower Limb—Toe Out Angle L, Lower Limb—Toe Out Angle L [SD], Lower Limb—Toe Out Angle R, Trunk—Transverse Range of Motion [SD], Turns—Steps in Turn [SD]
k-Nearest Neighbor	9	Lower Limb—Gait Cycle Duration L, Lower Limb—Single Limb Support R (%GCT), Lower Limb—Terminal Double Support R (%GCT), Lower Limb—Terminal Double Support R (%GCT) [SD], Lower Limb—Toe Off Angle L [SD], Lower Limb—Toe Off Angle R [SD], Lumbar—Coronal Range of Motion, Trunk—Coronal Range of Motion, Upper Limb—Arm Range of Motion L [SD]
SVM (linear kernel)	5	Lower Limb—Stride Length R, Lower Limb—Toe Off Angle R [SD], Lumbar—Transverse Range of Motion, Lumbar—Transverse Range of Motion [SD], Upper Limb—Arm Range of Motion R [SD]
SVM (rbf kernel)	24	Lower Limb—Double Support R (%GCT), Lower Limb—Foot Strike Angle R [SD], Lower Limb—Gait Speed L, Lower Limb—Gait Speed R, Lower Limb—Lateral Step Variability L, Lower Limb—Single Limb Support R (%GCT), Lower Limb—Stance L (%GCT), Lower Limb—Stride Length L, Lower Limb—Terminal Double Support R (%GCT), Lower Limb—Toe Off Angle L, Lower Limb—Toe Off Angle L [SD], Lower Limb—Toe Off Angle R, Lower Limb—Toe Off Angle R [SD], Lower Limb—Toe Out Angle R [SD], Lumbar—Coronal Range of Motion [SD], Lumbar—Sagittal Range of Motion, Lumbar—Sagittal Range of Motion [SD], Trunk—Coronal Range of Motion [SD], Trunk—Transverse Range of Motion [SD], Turns—Turn Velocity [SD], Upper Limb—Arm Range of Motion L, Upper Limb—Arm Range of Motion L [SD], Upper Limb—Arm Swing Velocity L [SD], Upper Limb—Arm Swing Velocity R [SD]
SVM (polynomial kernel)	12	Lower Limb—Circumduction R, Lower Limb—Elevation at Midswing R, Lower Limb—Foot Strike Angle L, Lower Limb—Foot Strike Angle R, Lower Limb—Gait Speed L, Lower Limb—Stride Length L, Lower Limb—Toe Off Angle L, Lower Limb—Toe Off Angle L [SD], Turns—Angle [SD], Upper Limb—Arm Range of Motion R, Upper Limb—Arm Range of Motion R [SD], Upper Limb—Arm Swing Velocity L
